# THE SITE OF AIRWAY OBSTRUCTION AMONG PATIENTS OF EMPHYSEMA: ROLE OF IMPULSE OSCILLOMETRY

**DOI:** 10.4103/0970-2113.44130

**Published:** 2008

**Authors:** HS Hira, Jitender Munjal, Sanjay Zachariah, MR Chauhan, Anshu Singh

**Affiliations:** 1 MD, DM, Professor of Medicine Chief, Pulmonary Medicine and Sleep Center, Maulana Azad Medical College and Lok Nayak Hospital, New Delhi - 110 002, India; 2 MD Resident, Maulana Azad Medical College and Lok Nayak Hospital, New Delhi - 110 002, India; 3 MD Senior Resident, Maulana Azad Medical College and Lok Nayak Hospital, New Delhi - 110 002, India; 4 Senior Technical Officer, Maulana Azad Medical College and Lok Nayak Hospital, New Delhi - 110 002, India

**Keywords:** Pulmonary function testing, FV Loop, Impulse Oscillometry, Flow Oscillation technique, Emphysema

## Abstract

**Objectives:**

To detect the site of airway obstruction in patients of emphysema by impulse oscillometry (IOS) and to compare its observatios with flow volume loop (FV loop) studies.

**Methods:**

Twenty-five patients of emphysema were subjected to both impulse oscillometry (IOS) and conventional spirometry. The resulting impedance spectra by IOS and FV loop were utilized to identify the site of airway obstruction in each of the patients. Both methods applied were also compared for sensitivity to identify airway and specify the site of obstruction.

**Results:**

Four patients were excluded from the final analysis as their impedance spectra showed significant upper airway influence, which would have made the localization of the site of airway obstruction by IOS invalid. Both IOS and FV loop could detect airway obstruction in all 21 patients. FV loop localized the site of obstruction as combined central and peripheral airways in 15 patients (71.4%) and as peripheral in 6 (28.6%).The IOS however, revealed the presence of the predominant site of obstruction peripheral in all the 21 patients, and both central and peripheral obstruction in 5 patients (23.8%).

**Conclusions:**

IOS had proven to be advantageous over FV loop studies as it could identify central and peripheral airway obstruction separately and established the predominant site of obstruction.

## INTRODUCTION

In emphysema the morphological basis of largely irreversible airflow limitation are varying combinations of inflammatory and fibrotic narrowing of peripheral airways^1^ and loss of elastic lung recoil with enhanced collapsibility of central airways.^2^,^3^ Studies suggest that the major site of airflow limitation in emphysema was peripheral airways.^1^

Impulse oscillometry is a method by which respiratory impedance can be measured simultaneously at various frequencies by means of a complex oscillation superimposed at the mouth during spontaneous quiet breathing.^4^ Conventional spirometry on the other hand is a well established method which produces characteristic and highly reproducible FV loop.^5^ In this study we evaluated and compared the site of airway obstruction in emphysema using both IOS and FV loop studies.

## METHODS

The study was conducted in the respiratory laboratory of department of pulmonary medicine of tertiary care referral hospital. Both Academic Board and Ethics committee of Institution approved the protocol. Informed written consent was taken from all the participants.

Clinical diagnosis of emphysema (n=25, 22 male and 3 female) was made in subjects on the basis of history of exertional dyspnea with scanty production of sputum, and physical examination revealing emphysematous chest. Chest radiographs showed hyper-inflated lung fields with peripheral vascular pruning and flattened diaphragms. Only patients with diffusion capacity of less than 70% of predicted value were selected. Those with low vital capacity, among whom diffusion capacity test was not possible, were subjected to CT Thorax to confirm the diagnosis of emphysema.

IOS and FV loop studies were performed in all included patients. All participants abstained from the medications for 24–72 hour before the tests. Patients with respiratory failure, acute exacerbations, acute respiratory infections (both upper and lower), chronic bronchitis, pulmonary tuberculosis and musculoskeletal or cardiovascular disease, or in whom chest skiagram showing additional lung disease, were excluded from this study.

### Measurement of lung functions

FV loop measurement was performed using Jaeger Masterscreen, Germany and was corrected according to body temperature and ambient pressure saturated with water vapor (BTPS) conditions.

IOS was performed with commercially available equipment (Masterscreen, Jaeger, Wurzburg, Germany and fulfilling the standard recommendations)^6^ consisting of an impulse generator, pneumotachograph and transducers. The impulse generator generates pressure pulses, with frequency range up to 100 Hz, at a rate of 3 pulses/second which are superimposed on the tidal breathing of the subject. Pressure and flow were recorded simultaneously at the mouth of the subject, by means of a Lilly type pneumotachograph (Erich Jaeger AG) connected to a differential pressure transducer (Sensym SLP004, +/− kPa). The common mode rejection ratio of both transducers was 70 dB. Calibration of pressure and impedance was performed with a reference impedance of 200Pa/L/S. Experimental data were analyzed using the impulse oscillometry software version LAB 4.33b on Windows 95 compatible PII 100 MHz personal computer.

During procedure of IOS, patient sat upright with head in neutral position, nose clipped, and keeping plastic mouthpiece tight between lips and teeth. Each subject was instructed to press his right hand against right cheek and left hand on the left cheek. After connecting to the equipment, the patient was allowed to breathe tidally. The measurement was started once the patient had minimum 3 stable breathing cycles. This measurement was made over a pre-set time of 150 sec. Out of the complete data set of each patient, which was displayed against time on computer screen, a series of as many regular tidal breaths as possible (minimum: a period of 30 s) were identified and marked for processing. Spectra of resistance (Rrs) and reactance (Xrs) were calculated by using a computer program. Frequency range used was 5Hz to 35Hz, which was relevant for diagnosis. Frequency lower than 5Hz is affected by spontaneous breathing and frequency higher 35Hz is less sensitive for diagnostic evaluation due to reduction in energy of IOS pressure flow signals. Output was obtained in a form of parameters R_5_, R_20_, R_35_, X_5_, X_35_ related to the predicted value, shown as percentages. Fres (resonant frequency), a frequency at which Xrs is zero, was calculated. The Zrs versus volume (ZV) graph was obtained to find air trapping. Resistance and reactance versus frequency spectra and visual interpretation graph (lung model) were also obtained.

### Interpretation of site of airway obstruction^7^

The central, peripheral and the predominant site of obstruction were determined using the impedance spectra and visual model of lung mechanics. The visual model of lung was based on Mead's 7-component model of lung mechanics. The impedance data obtained by IOS was square fitted using this model. The visual model of lung is divided into three parts: central, peripheral and oropharyngeal. Oropharyngeal part indicates compliance of cheeks and floor of mouth.

**Fig 1:**
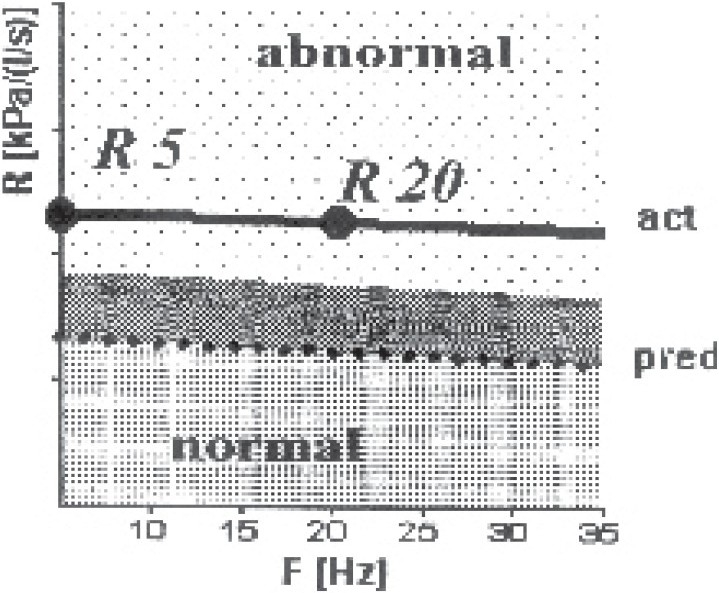
Impedance spectrogram of central obstruction of a patient showing resistance spectrum runs almost horizontally. The low-frequent resistance R5 and the higher frequent resistance R20 were on the same level.

**Fig 2:**
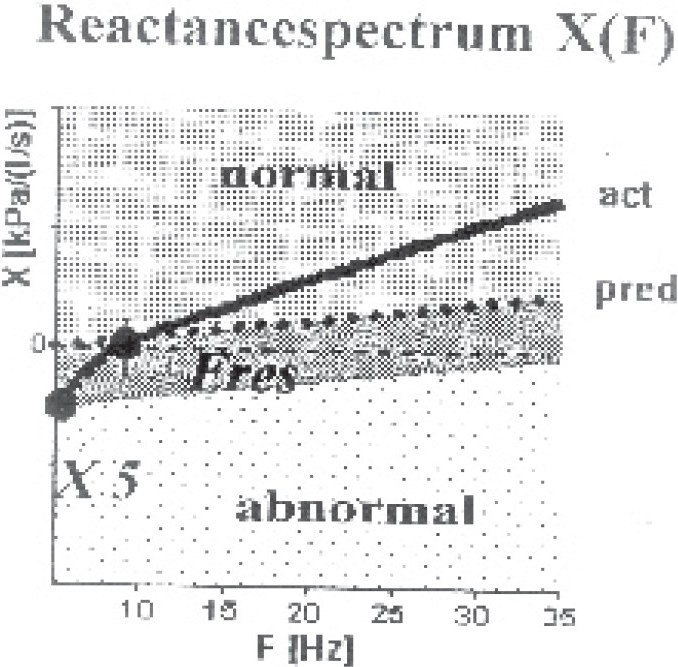
Reactance spectrogram of the same patient with Fig 1 showed reactance course X5 and the resonant frequency Fres in the normal range.

In central obstruction, the resistance spectrum was above the marked normal range (Fig 1). The resistance spectrum runs almost horizontally, i.e. the low-frequent resistance R5 and the higher frequent resistance R20 were on the same level. The reactance course and the resonant frequency were in the normal range (Fig 2). The measured R5 value was pathological if it exceeded 150% of its predicted value. The visual model of lung shows increased resistance in the central part.

**Fig 3:**
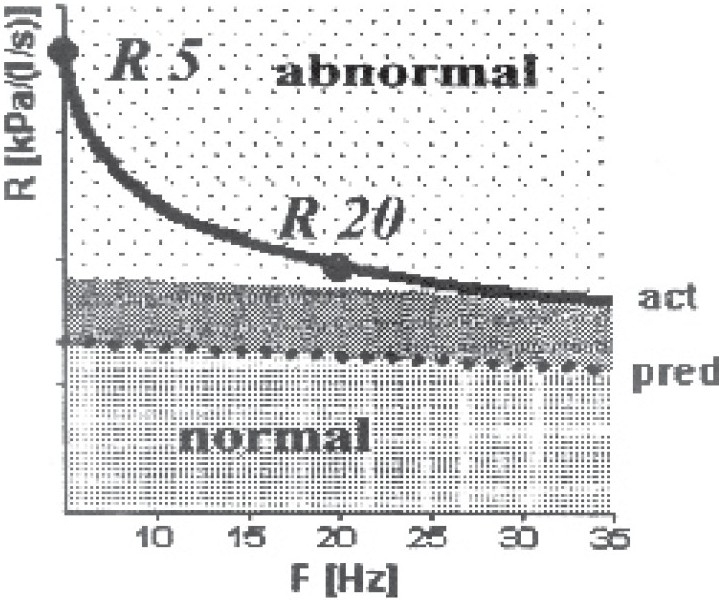
Resistance spectrum of a patient of peripheral obstruction was completely out of the marked normal range, the low frequency resistance R5 was clearly higher than the higher frequency resistance R20.

**Fig 4:**
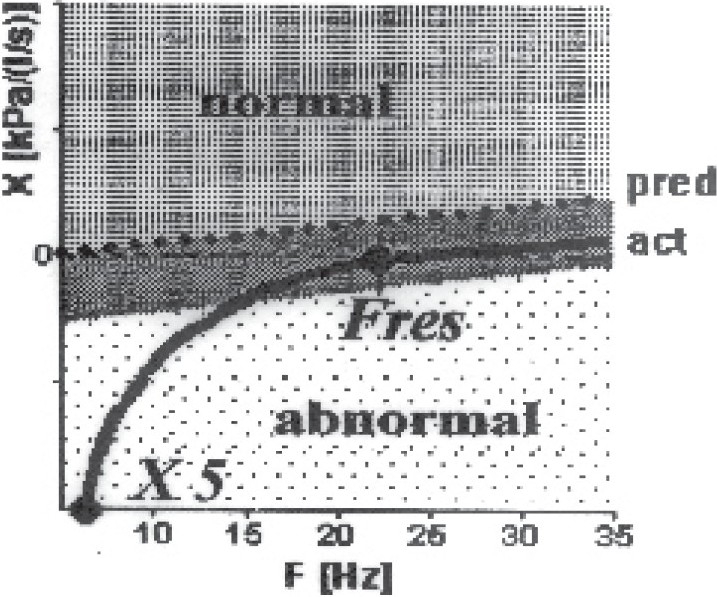
Reactance spectrum of the same patient of Fig 3 of peripheral obstruction demonstrated low frequency range X 5 considerably lower than the normal range and the resonance frequency Fres were increased.

In peripheral obstruction the resistance spectrum was completely or partly out of the marked normal range, the low frequency resistance R_5_ was clearly higher than the higher frequency resistance R_20_ (Fig 3). In contrast to the central obstruction, the reactance spectrum was in the low frequency range with X5 considerably lower than the normal range and the resonance frequency was clearly increased (Fig 4). The visual model of lung revealed increased resistance in the peripheral part.

**Fig 5:**
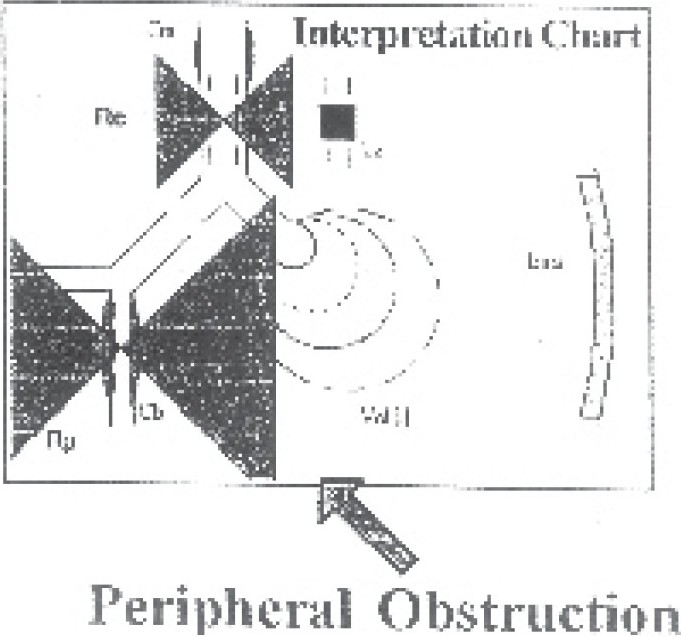
Interpretation chart (lung model) revealing peripheral obstruction Rp in a patient. Ers-elastance of thorax and lung; volume-volume of lung.

Predominant site of obstruction can be determined by looking at the visual model of lung. If central resistance was more than peripheral, then predominant site visible was in central airways. In case, peripheral resistance was greater than central resistance, the predominant site visualized was peripheral (Fig 5).

**Fig 6:**
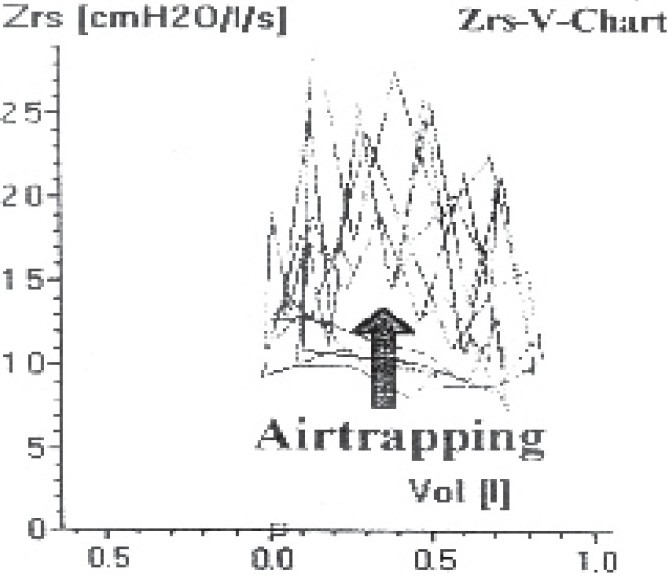
Z-V graph of a patient demonstrating the clear loops due to oscillometric impedance Zrs at 5Hz via the tidal volume; inspiration and expiration had a different course in the resistance hysteresis indicating air trapping in lung.

In IOS, Z-V graph (Fig 6) reflects the variability of airways within breath and this variability increases with increasing degree of obstruction indicating the air trapping.

### Flow Volume (FV) loop studies

FVloopstudies were carried out as per recommendations of American Thoracic Society.^8^ Forced expiratory spirometric data were obtained using a computerized system (Masterscreen Jaeger). Each subject performed at least three acceptable maneuvers and the standard spirometric parameters FV loop. FEV_1_, FEV_1_/VC, PEF, FEF_25_, FEF_50_, FEF_75_ were computed. All values were expressed as percentages of the predicted values. In addition to FV loop and calculation of forced vital capacity, slow (non-forced) expiratory vital capacity was calculated. Normally both values are identical but when they differ substantially (>0.2), suggests evidence of air trapping.

### Statistical analysis

All data was expressed as mean ± standard deviation. Presence and absence of central and peripheral obstruction in the patients was expressed as percentages and their significance was tested by Fischer Exact test (two tailed). Similarly, significance of predominant site of airway obstruction was tested by Fischer Exact test (two tailed). The comparison of IOS and spirometry parameters was done by student's t test. The relationship between FV loops with visual model of lung was tested by Fischer Exact test.

## RESULTS

This study included 21 patients (19 males and 2 females) of emphysema. Their mean age was 56 ± 18 years. The FV loop studies and impedance data (IOS) of the patients are summarized in Table 1.

**Table 1:** Parameters of Pulmonary Functions (FV Loop) and Impulse Oscillometry (IOS).

**Parameters**	**Values**
Age, yr	56 ± 18
Height, cm	163 ± 8
Weight, kg	43 ± 9
FVC, %predicted	60 ± 15.4
FEV_1_, %predicted	37.4 ± 15.8
FEV_1_/FVC, %predicted	60 ± 13
PEF, %predicted	42.5 ± 14
FEF_25_ %predicted	21.9 ± 16.6
FEF_50_ %predicted	14.9 ± 10.9
FEF_75_ %predicted	14.2 ± 10
Fres, Hz %predicted	31.9 ± 7.7
Zresp, %predicted	242.7 ± 141.8
R at 5 Hz, %predicted	200.3 ± 101.2
R at 20 Hz, %predicted	114.5 ± 40
R at 35 Hz, %predicted	121 ± 42.5
X at 5 Hz, kPa/l/s	−0.43 ± 0.28
X at 35 Hz, kPa/l/s	0.011 ± 0.07

FVC=Forced Vital Capacity; FEV_1_=Forced Expiratory Volume in 1st econd; PEF=Peak Expiratory Flow; FEF_25–75_ = Forced Expiratory Flow at 25%, 50%, 75% of vital capacity.

Fres=resonant Frequency; Zresp=Impedance of respiratory system; R5, R20, R35=Resistance at 5Hz, 20Hz, 35Hz;X5, X35=Reactance at 5Hz, 35 Hz.

*Values are mean ± standard deviation.

### Relationship between FV loop characteristics and site of airway obstruction detected by IOS

Of the 21 emphysema patients, FV loop detected presence of airway obstruction in all the patients (n = 21). Depending upon the characteristics of FV loop curves, patients were divided into two subgroups. In the subgroup E-1 (15) patients, FEV_1_ was decreased disproportionately to reduction of vital capacity, and FEF_25_, FEF_50_ and FEF_75_ were significantly reduced. FV loop revealed the presence of characteristic “kink”, which was presumably due to airway collapse and suggestive of obstruction of combined central and peripheral airways. IOS revealed presence of combined central and peripheral obstruction in only 5 (23.8%) same E-1 patients. Predominant site of obstruction detected by IOS was peripheral in all the 21 patients.

In subgroup E-2, (n = 6) FV loop revealed presence of airway obstruction without “kink” and the terminal portion revealed slow tail appearance of expiratory curve, characteristic of peripheral obstruction. IOS also revealed peripheral obstruction in these patients, and none of them had central obstruction.

IOS revealed peripheral obstruction in all the 21 patients. Central obstruction was noted in only 5 patients (23.8%), and even in these patients, the predominant site of obstruction was peripheral (Table 2).

**Table 2:** Site of airway obstruction by impedance spectra and visual model of lung of IOS among 21 patients of emphysema.

Central obstruction	Number of patients
Present	5 (23.8%)
Absent	16 (76.2%)
Predominant	0 (0%)

Peripheral obstruction	Number of patients

Present	21 (100%)
Absent	0 (0%)
Predominant	21 (100%)

Air trapping was evident in 16 (76%) patients by spirometry, and in 20 (95%) patients by IOS (Fig 6). Although IOS detected air trapping in more number of cases but this was not statistically significant.

## DISCUSSIONS

In last three decades, there has been much interest in recognizing disease of the peripheral airways less than 2 mm in diameter. Early studies indicated that the peripheral airways contributed only 10% of total airway resistance^9^ but recent studies showed that they contributed about 30% of the total airway resistance.^10^ Hence there could be moderate increase in the resistance of the peripheral airways with little detectable effect upon expiratory flows. Several methods have been developed to identify early disease of the peripheral airways but for the most part they are too complex for routine testing.^11^ It may be possible, however, to identify the peripheral airways involvement by FV loop, if the expiratory flow at low lung volumes was carefully analyzed.^12^ The peripheral airways lack cartilaginous support for their patency. Their patency depends upon the elastic recoil. Since the recoil pressure diminishes during decreasing lung volume, the resistance of small airway increases relative to that of central airways in the terminal part of the forced expiration. Therefore, peripheral airway disease can be suspected if the FEV_1_ and PEF are normal, whereas measurements obtained at low lung volumes are abnormally reduced and give ‘slow tail’. The FEF_25–75%_ has been recommended as a good way to identify small airway disease^13^ but measurements obtained from the terminal part of expiration such as FEF_75%_ or FEF_75–85%_ should be more sensitive.^14^ However, these measurements are very much influenced by the patient's effort, which limits their clinical usefulness.

In advanced airway obstruction, both FEV_1_ and FVC are reduced with decreased overall air flows, with a further decrease at low lung volumes. This is thought to indicate involvement of more proximal airways i.e. central airways.^5^ In them, relatively high flow is maintained during first 15–25% of expired volume and then there is an abrupt fall to reduce airflow over the rest of the breath. Therefore, when both central and peripheral airways are obstructed, FV loop cannot evaluate the predominant site of obstruction.

On the other hand, IOS is an effort independent technique that can not only detect central and peripheral obstruction separately but also the predominant site of air obstruction by visual model of lung.^7^ To compare the observations of both FV loop and IOS, 21 patients of emphysema were divided to two groups.

In group E1 of 15 emphysema patients (71.4%), FV loop revealed a characteristic “kink” in the early portion of the forced expiratory part of flow-volume loop. This spirographic kink is a sign of emphysema.^15^ The kink, which is presumably due to airway collapse, is facilitated by destruction of lung parenchyma, which results in loss of elastic recoil, a positive pleural pressure, high bronchiolar resistance, and structural weakness of the major airways. This inflection represents the onset of the marked dynamic compression of the intrathoracic airways during the maneuver to record FV loop. Therefore, whether the peripheral airways were severely diseased or not, could not be predicted by FV loop since there was superimposed central airway collapsibility. Additionally, it could not indicate the predominant site of obstruction. However, IOS was able to detect presence of combined central and peripheral obstruction in 5 of 21 of participated patients and predominant site identified in all of them was peripheral. This could be because IOS was recorded during quiet breathing while FV loop required maximal respiratory effort, hence dynamic compression of central airways was more evident during FV loop recording. This finding confirmed that the ‘kink’ or airway collapse is a dynamic phenomenon in emphysema and presence of it indicates severe lung parenchyma destruction.

In group E-2 of 6 patients, FV loop revealed ‘slow tail’ appearance and the kink was absent. These patients presumably had less severe form of emphysema. The IOS revealed only peripheral obstruction in all of them and none had central obstruction. This explained that emphysema was an essentially a peripheral airway disease and latter were diseased early in the course of disease. Various earlier studies in smokers revealed that forced oscillation technique failed to reveal early emphysema in smokers and low diffusion capacity is the best indicator of early emphysema.^16^ In our study, by virtue of selection, emphysema patients had DLCO of less than 70% of the predicted. Therefore, it is not possible to compare whether IOS or DLCO diagnosed emphysema early.

The characteristic resistance and reactance curves of IOS, which were used in the clinical interpretation, are affected by the upper airway shunt. The plateau in the reactance course of the impedance spectra indicates upper airway shunt. Therefore, 4 of 25 patients selected initially were later excluded from the final evaluation due to presence of this upper airway shunt them. From recent studies it has become clear that a precise correction of shunt properties of upper airway was a prerequisite for reliable analysis of the site of airway obstruction; the negative frequency dependence of Rrs observed in patients with airflow obstruction was based on the shunt properties of the upper airways.^17^ Part of the flow generated by the loudspeaker does not enter the lower airways but is lost in motions of the upper extrathoracic airways, most notably the cheeks. This results in an error in the estimation of respiratory impedance. This upper airway artifact tends to exaggerate the negative frequency dependence of Rrs and decrease the imaginary part of impedance. Supporting the cheeks with the palms of the hands (tried in our subjects also) does not fully eliminate this error.

Michaelson et al^18^ showed that the residual error may still be quite large and it is preferable to also measure upper airway wall impedance and to correct data for it. Peslin et al^17^ used head plethysmograph that permits the measurement of upper airway wall motion forced oscillation studies and they concluded that unless the upper airway compartment was taken in account, they seriously impair interpretation of the impedance curves in terms of clinical physical properties of the respiratory systems, using more or less sophisticated models. Farre et al^19^ concluded that using the absolute or relative change in oscillatory admittance as an index for assessing the bronchial reactivity eliminates, or markedly reduces, the upper airway artifact when using the forced oscillation technique.

Contrary to continuous test signals like sine waves or pseudo random noise, used in earlier techniques, which create a discontinuous spectrum, the impulse oscillometry (discontinuous test signals) generates a continuous spectrum. With the help of this continuous spectrum, the differentiation of upper airway obstructions becomes possible. It has been shown that plateau in the reactance course of oscillometry differentiates extrathoracic influence on the impedance data. This fact was not realized at the beginning of the present study.

Impulse oscillometry gives a visual display of air trapping in form of opened loops in Z-V graph. However, in FV loop studies, both forced and slow vital capacities are required to indicate the presence of air trapping. The difference between slow and forced vital capacity is mainly due to phenomenon of dynamic compression resulting from increased resistance of intrathoracic airways and loss of elastic lung recoil. When marked, it suggests pulmonary emphysema but it can also be seen in uncomplicated bronchospasm in asthma. IOS detected air trapping in 20 out of 21 participants of this study; however, this did not reach a statistically significant level.

In conclusion, both IOS and FV loop detected airway obstruction in all emphysema patients. Both techniques had their own characteristics and limitations and both provided, each by their own typical characteristics, information about mechanical behavior and properties of respiratory system. Unlike FV loop, IOS identified central and peripheral airway obstruction separately even when both were operative simultaneously, and determined the contribution made by each component towards total airway obstruction. Additionally, IOS in contrast to FV loop was an effort independent, less time consuming and required minimal patient cooperation.
